# Changing Trends in Suicidal Ideation and Its Influencing Factors During the Transition From Quarantine to Post-Quarantine Among Chinese University Students During the COVID-19 Surge: Six-Wave Panel Study

**DOI:** 10.2196/74370

**Published:** 2025-09-05

**Authors:** Lijing Li, Tingzhong Yang, Sihui Peng, Randall R Cottrell

**Affiliations:** 1 Department of Obstetrics and Gynecology, Yongkang Women and Children’s Health Hospital, Yongkang, China; 2 Maternal and Child Healthcare Service Department, Yongkang Women and Children's Health Hospital, Yongkang, China; 3 Research Center for Digital Health Behavior Theory and Management, Zhejiang University National Health Big Data Institute, Hangzhou, China; 4 Injury Control Research Center, West Virginia University, Morgantown, WV, United States; 5 Department of Public Health and Preventive Medicine, School of Medicine, Jinan University, No. 601 Huangpudadao West, Guangzhou, 510632, China, 86 02085220267; 6 Public Health Studies Program, School of Health and Applied Human Sciences, University of North Carolina Wilmington, Wilmington, NY, United States

**Keywords:** COVID-19, lockdown, suicide, uncertainty, stress

## Abstract

**Background:**

To mitigate the rapid spread of COVID-19, numerous countries have adopted lockdowns and quarantine measures. Despite their public health benefits, the effects of these measures on suicidal ideation have not been well documented.

**Objective:**

This study aims to examine the relationship among COVID-19 infection, perceived beliefs, uncertainty stress, and suicidal ideation during the transition from quarantine to post-quarantine periods amid China’s COVID-19 surge.

**Methods:**

A prospective longitudinal observational design was used. Changing trends across the 6 time points were assessed using the Mann-Kendall test and the Cochran-Armitage test. A generalized estimating equation was used to analyze the associations between independent variables and suicidal ideation.

**Results:**

A total of 221 (96.5%) participants completed all 6 observation waves. The prevalence of suicidal ideation during the quarantine period was 16.7% (n=37), 14.5% (n=32), and 14.5% (n=32), while during the post-quarantine period, it was 13.8% (n=30), 10.9% (n=24), and 10.0% (n=22), respectively. A significant downward trend in suicidal ideation was observed. In contrast, perceived risk, perceived severity, and the number of new infections exhibited significant upward trends (*z* scores of 9.56, 7.13, and 3.69, respectively; *P*<.001, *P*<.001 and *P=*.002, respectively). However, uncertainty stress remained stable over time (*z*=0.71; *P*=.48). The generalized estimating equation indicated that perceived risk (β=0.5482; *P*<.001), perceived severity (β=0.0817; *P*=.007), and uncertainty stress (β=0.1776; *P*<.001) were positively associated with suicidal ideation. The number of new infections (β=0.0041; *P*=.49) was not significantly associated with suicidal ideation.

**Conclusions:**

This study found that suicidal ideation gradually declined following the lifting of quarantine measures. Perceived risk, perceived severity, and uncertainty stress, rather than numbers of infected cases, were significantly associated with suicidal ideation. These findings highlight the importance of addressing individuals’ perceptions with real-world context when developing effective strategies to manage COVID-19 and future infectious disease outbreaks.

## Introduction

The COVID-19 pandemic is a global public health emergency that poses a great threat to people’s health and causes large disruption to people’s lives and work. In order to curb the rapid spread of COVID-19, many countries have implemented “lockdown” policies [1-4]. “Lockdown” is an emergency protocol that prevents the public from moving from one area to another. In China, complete and partial “lockdowns” were also implemented in some cities [5,6]. These public health emergency measures effectively prevented the rapid growth of the COVID-19 infection and greatly limited the epidemic of the disease in China [6].

While lockdown measures are crucial in containing the highly infectious COVID-19 virus, they can also have a negative impact on mental health. Quarantine, regardless of the cause, has been associated with various mental and behavioral problems [3,4,7], including those related to the COVID-19 situation [1,3-5]. A review reported that quarantine revealed a positive association with posttraumatic stress disorder, anxiety, and depression [8]. Some studies have also found a link between quarantine and subsequent anxiety and depression [9,10]. A study conducted in China showed that personal quarantine increased anxiety, fear, and anger among individuals [5]. However, these studies primarily relied on cross-sectional observations, making it challenging to establish causal relationships. To address this gap in the literature, we investigate the changing trends of mental health issues and their associations during the transition from quarantine to post-quarantine periods in the COVID-19 epidemic in China.

According to the Stimulus-Cognition-Mental Health (SCM) theory, any stimulus may lead to an awareness of the serious threat posed by the disease (cognition), which in turn can trigger mental responses, ultimately resulting in mental and behavioral problems [11,12]. In the context of personal quarantine and its impact on mental and behavioral problems, the research framework should encompass stimuli, perceived beliefs, and mental health issues, facilitating the identification of causal relationships.

Obviously, the quarantine is a direct stimulus in the theory in this study, which may induce mental response, uncertainty stress, perceived risk, and perceived severity of COVID-19, ultimately impacting people’s mental health issues [13]. Many studies found that quarantine is positively associated with mental and behavioral problems [4,5,8-10]. This study’s focus is on how quarantine affects mental problems through mental response. Another stimulus is new COVID-19 infection in the period of observation. When people are infected during observation, it is a great shock to them, undoubtedly leading to a strong mental response, which can lead to mental disorder. It is inevitable that people would experience dual stimuli, quarantine, and COVID-19 infection in this study. Our task is to observe their respective roles in the SCM model.

This study hypothesizes that both quarantine and COVID-19 infection act as a direct stimuli, which may induce perceived risk and perceived severity of COVID-19, uncertainty stress, ultimately impacting people’s suicidal ideation [13]. Many studies reported that quarantine and COVID-19 infection influenced mental and behavioral problems [1,3,5,7,9], but these studies only included stimuli (quarantine or COVID-19 infection) and outcome variables (mental problems) in their research framework. This study will include more aspects, stimuli, perceived beliefs and uncertainty stress, and mental problems.

According to the Health Belief Model, the health behavior of every individual is motivated and influenced by core belief variables [11,12]. Previous studies have demonstrated a close association between strong perceptions of risk and threat associated with COVID-19 and mental and behavioral outcomes [13-16]. In this study, we examine individuals’ perceived risk and perceived severity of COVID-19 during quarantine.

Festinger [17] hypothesized that uncertainty can lead to cognitive dissonance. When individuals are confronted with intense and prolonged uncertainty stress, it can have detrimental effects on their mental and behavioral well-being [18]. The COVID-19 pandemic is characterized by a high degree of uncertainty, as it emerged suddenly, spread rapidly, and then declined abruptly. These factors contribute to a significant level of uncertainty stress. Numerous studies have found a positive association between uncertainty stress and mental and behavioral problems during the COVID-19 pandemic [18-21]. Some studies have specifically highlighted higher levels of mental stress among individuals who were under quarantine [22,23], although few have directly examined the role of uncertainty stress. In addition, for individuals who were not under quarantine, Vandoros and Kawachi [24] found a positive association between economic uncertainty and suicidal ideation. Another study demonstrated a link between uncertainty stress and suicidal thoughts [25].

The concentrated and prominent manifestation of mental problems is suicide, which is closely related to mental stress, anxiety, and depression across many populations [26-28]. It would be established that there is an association between quarantine and suicide, as well as other mental health problems [1,3,5]. However, so far, no studies have examined this relationship. Although suicidal ideation may have a different underlying mechanism than actualized suicide, it is the most proximate precursor and a necessary condition for suicide [27,29]. In this study, we aim to examine the status of uncertainty stress during quarantine in the COVID-19 surge in China and its influence on suicidal ideation. By investigating uncertainty stress as a specific dimension of the overall stress experienced during quarantine, we can gain a deeper understanding of its impact on individuals’ mental well-being and the potential risk of suicidal ideation.

## Methods

### Study Design

A prospective longitudinal observation study was designed to examine temporal trends and changes in suicidal ideation over a 6-week period of time and their associations with selected stimulus and support variables from the quarantine to the post-quarantine period among university students during the COVID-19 surge in China.

### Setting

This study was conducted during the COVID-19 lockdown period in Guangzhou, China, with a specific focus on university students who were under quarantine. These students were confined to their residential buildings and were not permitted to move freely in or out of these areas. Lockdown measures began on October 24, 2022, affecting all areas of Haizhu District and parts of Panyu and Tianhe Districts. Restrictions were gradually lifted starting on November 27, 2022, in Haizhu and Tianhe, with the entire city of Guangzhou fully released from lockdown by November 30. Notably, university campuses did not immediately lift restrictions but instead gradually eased them over 1 week.

Our observation started in later stages after the lockdown was implemented, which may capture the cumulative mental effects of the lockdown. This panel study included 6 waves of data, which were collected over 6 weeks. Wave 1 (November 19, 2022), Wave 2 (November 24, 2022), and Wave 3 (December 1, 2022) are quarantine periods; Wave 4 (December 8, 2022), Wave 5 (December 16, 2022), and Wave 6 (December 23, 2022) are post-quarantine periods.

### Participants

Participants were recruited through a survey advertisement on the campus Bulletin Board System, a social media platform at the university used for both public announcements and internet-based social interaction. Inclusion criteria included the following: (1) quarantine university students, (2) having access to a smartphone, (3) knowing the Chinese language, and (4) being willing to participate in the panel study and provide follow-up information at 6 scheduled observation points. Participants were excluded if they refused to provide this information or had a medical condition that could limit or preclude their participation.

Within the registration system, potential participants were screened to ascertain eligibility. Upon consent, participants received an electronic questionnaire and instructions on how to proceed. After reading the instruction, they were asked to provide an electronic consent by tapping the “Confirmation and Authorization” button to opt in. After authorizing the consent, they were directed to the questionnaire and began answering the survey.

A special administrative WeChat (Tencent) group was established to manage the follow-up data collection, using a unique QR code for each respondent. The QR code was the vehicle, not only for identifying unique participants but also for prohibiting nonparticipants from taking the survey. After scanning the QR code, survey participants could enter the investigation group without further preconditions. All responses were anonymous. The questionnaire took 10 minutes to complete, and the same survey protocol was used for each wave of the survey to ensure homogeneity of data administration and collection.

### Measurement

#### Dependent Variable

The self-report suicidal ideation measure used in this study was based on the response to the following question: “Did you seriously consider suicide during the past year?” Options were dichotomous: “yes” or “no.” “No” was coded as 1, and “yes” was coded as 2 [21,27].

#### Independent Variables

##### Demographics

Age, gender, ethnicity, major, parental occupation, and personal monthly spending.

##### Perceived Risk and Perceived Severity of COVID-19

They came from 2 key concepts in the Health Belief Model [11,12]. Perceived risk was measured by the question, “Do you feel you are always at risk of being infected with COVID-19?” Perceived severity was measured by a statement: “Infection with COVID-19 has serious health consequences.” Responses were on a 5-point Likert-type scale ranging from “strongly disagree” to “strongly agree.”

##### Uncertainty Stress

Uncertainty stress was measured using a scale designed by Yang et al [30], which has demonstrated acceptable validity and has since been used extensively in Chinese research [30,31]. It covered 4 items: current life uncertainty (“life is unstable and cannot be controlled”), social change uncertainty (“uncertain about what will happen in the future”), goals uncertainty (“uncertain about how to achieve goals”), and social values uncertainty (“cannot follow social values”).

Respondents rated these items on a 5-point scale from feel no stress (0), a little stress (1), some stress (2), and considerable stress (3) to very strong stress (4). A total stress score was obtained by adding up the responses to the individual questions. The higher the total score, the greater the perceived level of the uncertainty stress [30,31]. In this study, we estimated the reliability and validity of uncertainty stress questionnaire at the 6 different observation time points. The Cronbach α coefficient was 0.85, 0.89, 0.88, 0.89, 0.90, and 0.90, respectively, suggesting acceptable reliability. Fact analysis was conducted in the following steps. For the Kaiser-Meyer-Olkin measure of sampling adequacy, the lowest of the values at each of the 5 survey waves was 0.85. The Bartlett test of sphericity for all waves was significant (*P*<.001), suggesting the samples were factorable. One factor was extracted at each wave, accounting for 68% (the lowest value in the 6 waves) of the variance. The lowest value of factor loading of these items was 0.68, 0.66, 0.76, and 0.62 at the 6 observation time points, indicating good construct validity.

### Data Analysis

All data were entered into a database using Microsoft Excel. They were then imported into SAS (version 9.4; SAS Institute Inc) for the statistical analysis. As most of the continuous variables included in this study were not normally distributed, we used nonparametric testing methods to examine the changing trends. The Mann–Kendall test was used to assess changing trends across the 6 observation points, with the parameters for statistical testing being the *z* value [32,33]. For categorical variables, the Cochran-Armitage test was used to examine their changing trends; the parameters for statistical testing were also the *z* value [34]. The generalized estimating equation was used to examine the association among confirmed new COVID-19 infected persons, behavioral beliefs, uncertainty stress, and suicidal ideation [35].

### Ethical Considerations

The study was conducted following the Declaration of Helsinki and was approved by the ethics committee of the Medical Center at Jinan University (JNUKY-2022‐047). All participants received an electronic instruction on how to proceed. After reading the instructions, they were asked to provide electronic consent by tapping the “Confirmation and Authorization” button and then were directed to the questionnaire. Participation in the study was entirely voluntary, with participants having the right to withdraw or discontinue at any time without any penalties or loss of benefits. All information was kept strictly confidential and anonymized to ensure participants could not be identified. As appropriate, a token of appreciation, 35 RMB (approximately US $5.00) were given to those participants who completed all 6 questionnaires.

## Results

We recruited 229 participants at baseline. The baseline data were linkable to all subsequent questionnaire administrations, 4 intermediate waves, and a final observation point. A total of 221 (96.5%) remained for all repeated measures. The majority of respondents were Han Chinese (n=215, 97.3%) and 76.5% (169/221) were female. In terms of age, 13.6% (30/221) were younger than 20 years old, 21.7% (48/221) were aged 22 or older, and the remaining respondents were between 20 and 22 years old. Of the participants, 62.3% (131/221) were majoring in science, engineering, and medicine while 37.7% (79/221) were majoring in the humanities and social sciences (Table 1).

**Table 1.** Sample characteristics.

Group	Values, n (%)	
Age (year)		
<20	30 (13.6)	
20	54 (24.4)	
21	50 (22.6)	
22	39 (17.6)	
22 and older	48 (21.7)	
Ethnicity		
Han	215 (97.3)	
Minority	6 (2.7)	
Sex		
Male	52 (23.5)	
Female	169 (76.5)	
Monthly expenditure (RMB*)^a^*		
<1500	61 (27.6)	
1500-2000	67 (30.3)	
2000-2500	47 (21.3)	
2500 and more	46 (20.8)	
Major		
Science and engineering, and medicine	131 (62.3)	
Humanities and social sciences	79 (37.7)	
Father’s education level		
Primary school and low	33 (14.9)	
Junior school	63 (28.5)	
High school	51 (23.1)	
Junior college	33 (14.9)	
College and more	41 (18.6)	
Father’s occupation		
Operation	143 (64.7)	
Administration, commercial, and service	50 (22.6)	
Science, technology, and education	12 (5.4)	
Others	16 (7.2)	
Mother’s education level		
Primary school and low	56 (25.3)	
Junior school	59 (26.7)	
High school	47 (21.3)	
Junior college	26 (11.7)	
College and more	33 (14.9)	
Mother’s occupation		
Operation and others	132 (59.7)	
Administration, commercial, and service	39 (17.6)	
Science, technology, and education	26 (11.8)	
Others	24 (10.9)	

a 1 RMB=US $0.14.

**Figure 1.**
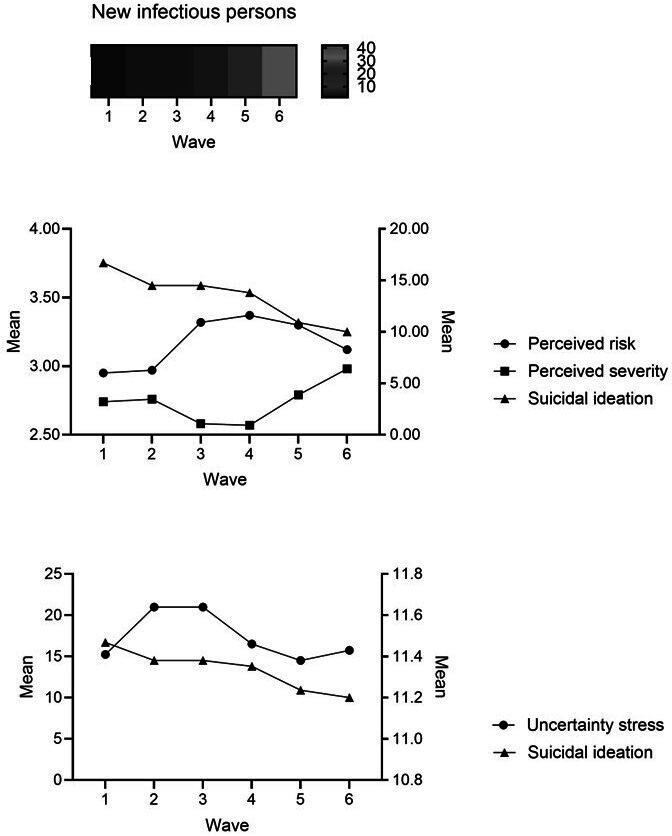
Time change trend in all variables.

The prevalence of suicidal ideation was 16.7% (95% CI 12.5%-20.9%), 14.5% (95% CI 10.0%-19.3%), and 14.5% (95% CI 9.8%-19.2%) in the quarantine period and 13.8% (95% CI 9.5%-18.3%), 10.9% (95% CI 6.8%-15.0%), and 10.0% (95% CI 6.0%-14.1%) in the post-quarantine period. The Mann-Kendall test estimating suicidal ideation found significant changes (*z*=−4.06; *P*<.001). Simultaneously, perceived risk and perceived severity showed a statistically significant upward trend across the total observation period, with a *z* value in the former of 9.56 and 7.13 for the latter, both at *P*<.001, and this was also true for new infectious persons (*z*=3.69; *P*=.002), with the prevalence being 1.7%, 3.5%, 3.5%, 4.6%, 17.2%, and 42.0% at the time of each wave, respectively. However, uncertainty stress did not change significantly over the observation period (*z*=0.71; *P*=.48; see Table 2 and Figure 1).

**Table 2.** Time change trend in suicide ideation, perceived risk, perceived severity, uncertainty stress, and new infectious persons.

	Suicidal ideation prevalence, proportions (95% CI)	Perceived risk, mean (95% CI)	Perceived severity, mean (95% CI)	Uncertainty stress, mean (95% CI)	The number of new infectious patients, proportions (95% CI)
Wave					
1	16.7 (12.5-20.9)	2.95 (2.82-3.06)	2.74 (2.62-2.86)	11.41 (10.95-11.87)	1.70 (0.50-4.57)
2	14.5 (10.0-19.3)	2.97 (2.85-3.09)	2.76 (2.65-2.88)	11.64 (10.88-11.82)	3.50 (1.58-7.01)
3	14.5 (9.8-19.2)	3.32 (3.20-3.43)	2.58 (2.47-2.68)	11.64 (11.18-12.10)	3.50 (1.58-7.01)
4	13.8 (9.5-18.3)	3.37 (3.26-3.49)	2.57 (2.46-2.69)	11.46 (10.98-11.94)	4.60 (2.19-8.16)
5	10.9 (6.8-15.0)	3.30 (3.18-3.41)	2.79 (2.67-2.90)	11.38 (10.90-11.87)	17.20 (12.46-22.83)
6	10.0 (6.0-14.1)	3.12 (3.00-3.24)	2.98 (2.86-3.11)	11.43 (10.94-11.90)	42.00 (35.49-48.89)
Overall prevalence or mean	13.6 (11.9-25.3)	3.14 (3.00-3.19)	2.74 (2.86-3.11)	11.49 (10.98-11.92)	12.08 (6.03-19.17)
Time trend test (*z* value)	−4.06^a^	9.56^a^	7.13^a^	0.71	3.69^a^

a *P*<.01.

Table 3 shows the relationship among perceived risk, perceived severity, uncertainty stress, the number of new infectious patients, and suicidal ideation while accounting for repeat observations within individuals. It indicates that perceived risk and perceived severity were positively associated with suicidal ideation, with a β of 0.5482 (*P*<.001) and 0.0817 (*P*=.007) respectively. Higher uncertainty stress was associated with higher suicidal ideation (β=0.1776; *P*<.001). The number of new infectious patients did not have an association with suicidal ideation (β=0.0041; *P=*.49).

**Table 3.** Factors associated with suicidal ideation.

Group	β (SE)	*P* value
Perceived risk	0.5482 (0.0326)	<.001
Perceived severity	0.0817 (0.0303)	.007
Uncertainty stress	0.1776 (0.0207)	<.001
The number of new infectious patients	0.0041 (0.0059)	.49

## Discussion

### Contributions to the Literature

There is little evidence on changing trends in the impact of quarantine measures on suicidal ideation. In this study, the decline in suicidal ideation did not occur immediately after the lifting of quarantine measures; instead, a delayed effect or “grace period” was observed. Uncertainty stress was consistently high throughout the observation period and positively associated with suicidal ideation.

### Principal Findings

This study aimed to fill a research gap by examining the changing trends of suicidal ideation and related factors during the transition from quarantine to post-quarantine periods in the COVID-19 surge in China. The prevalence of suicidal ideation was found to be 16.7%, 14.5%, and 14.5% during the quarantine period and 13.8%, 10.9%, and 10.0% during the post-quarantine period, showing a significant downward trend over the entire observation period. These findings indicate that quarantine has an impact on suicidal ideation. It is important to note that the decline in suicidal ideation did not occur immediately after the release of quarantine measures, but rather there was a grace period. Particularly in the early stages of release, the prevalence of suicidal ideation remained relatively high, with a larger decline observed in the later period. This suggests a time-lagged effect of lockdown on influencing suicidal ideation, a common phenomenon in public health policies. Understanding the duration of this lag can assist in predicting and addressing the consequences of implementing and lifting lockdown measures. These findings also highlight the necessity of expanding mental health services to individuals in quarantine.

Perceived risk and perceived severity showed a statistically significant upward trend across the total observation period. This means that the quarantine measure has decreased the role of perceived risk and perceived severity. This study adds new evidence on the influence of quarantine on the perceived risk of contracting COVID-19 and the perception of its severity. This phenomenon may be explained by SCM theory. Quarantine is a protective factor against stress stimuli, which can decrease high levels of perceived risk and perceived severity. When people are quarantined, they may think they are safe. However, when they are released, they may feel they have lost protection and perceive more risk and severity for COVID-19 infection.

Uncertainty stress is an important observational variable in this study. This study showed that uncertainty stress did not significantly change, remaining at a high level throughout the observation period. Extreme uncertainty is a key issue in the COVID-19 epidemic, which includes the uncertainty of COVID-19 itself and the suddenness of the lockdown and release policy; these factors may lead people to a high uncertainty stress level. Many studies found that uncertainty stress was positively associated with mental and behavioral problems during the COVID-19 period [18-21]. Due to uncertainty stress closely associated with social trust, it is possible that it influences the implementation of the policy during the COVID-19 pandemic [36-38]. We believe that sustainable high-level uncertainty stress poses a large challenge for people’s compliance with COVID-19 control policies.

The Health Belief Model argues that individual behavior is determined by perceived risk and perceived severity [11,12]. The risk of disease and the severity of outcomes are crucial predictors of mental problems [11,12]. This study found that perceived risk and perceived severity of COVID-19 were positively associated with suicidal ideation. Many studies found there is a close association between strong risk and threat perceptions of COVID-19 and mental and behavioral outcomes [13-16]. At the same time, some studies also indicate that suicide is a prominent manifestation of mental problems [26-28]. Therefore, it is rational that perceived risk and perceived severity are related to suicidal ideation.

This study found that uncertainty stress was positively associated with suicidal ideation. A host of evidence supports the assertion that uncertainty constitutes a powerful stressor [12,39,40], significantly associated with many mental and behavioral problems related to COVID-19 [18,21]. An outstanding characteristic of the COVID-19 epidemic is uncertainty. It is a new virus, and much of the information about it is unknown or incomplete, with considerable ambiguity surrounding the disease, and there are frequent changes in pathogens. It should be emphasized that this characteristic has persisted so far. It should be emphasized that uncertainty is also closely related to the government’s way of disease control, such as ambiguity in providing information, the suddenness of implementing lockdown and opening up, inconsistencies in policies issued, low professionalism and authority of experts invited, and other factors. A basic strategy to decrease uncertainty is to implement scientific decisions. All policies and interventions need to be based on scientific evidence when dealing with the COVID-19 epidemic. However, in some areas or regions, policies and interventions were not based on scientific evidence. Many of these policies and interventions were arbitrary and ineffective. Ineffective policies and interventions can cause people to question their government, which ultimately may be detrimental to disease control.

Suicidal ideation decreased over the study period, despite upward trends in perceived risk, perceived severity, and the number of new patients. However, perceived risk was positively associated with suicidal ideation, and higher uncertainty stress contributed to higher suicidal ideation. This phenomenon may be difficult for some people to understand. It should be noted that trend analysis provides a description of the changes in variables during the observation period, whereas correlation analysis of the observed research variables examines the interactions between multiple factors, considering individual differences and temporal changes. Therefore, the results from trend analysis and correlation analysis should not be expected to align perfectly. This study also performed subgroup analysis based on different sample characteristics, which yielded results consistent with the previous ones. This phenomenon is observed in many other studies [41-43].

This study found a significant increase in the number of new infectious individuals throughout the observation period, which aligns with real-world observations. When the lockdown was lifted, people regained access to the outside world, increasing opportunities for infection, which led to a rise in the number of patients [8]. While quarantine measures were linked to suicidal ideation, the increase in new infections did not show a similar association with suicidal ideation. This highlights the importance of carefully considering the mental health impacts of quarantine when designing lockdown policies.

### Study Limitations

There are several limitations to this study. First, our sample size appears to be small. Nevertheless, this is a prospective longitudinal panel study, and the variables included were repeatedly measured for each participant, which ensured statistical power for the tests used in this study was high. We estimated the power for repeated measures for each variable [44]. This analysis calculated statistical power (1-β) at given sample size (N=221), 5% significance level, and effect size used as a parameter in the model. A statistical power of 0.8 and higher was considered acceptable [45]. Since the effect size of various variables may differ, their statistical powers are also different. The results showed that statistical powers for all variables achieved accepted level (1-β=0.8 and higher). This indicates that the sample size in this study was large enough to make appropriate inferences. Second, sample attrition may introduce “cluster” bias because many longitudinal studies likely overrepresent some of these characteristics. A more sophisticated design and more representative sample would be necessary to resolve this problem. Another important limitation is that our participants were restricted to university students, who were selected due to their good cooperation in follow-up for a panel study. Thus, our results cannot be generalized to the wider Chinese population.

### Conclusion

This study found that suicidal ideation among Chinese university students declined over time as quarantine measures were lifted, but the decrease was delayed, indicating a lagged mental health effect of the lockdown. Perceived risk and perceived severity of COVID-19 increased, suggesting that sense of safety during lockdown may have reduced risk perception, which then rebounds once restrictions are lifted. Uncertainty stress remained consistently high throughout, highlighting a persistent mental health risk burden during the COVID-19 surge. Moreover, higher perceived risk, higher perceived severity, and higher uncertainty stress were related to a higher likelihood of reporting suicidal ideation. However, the proportion of infected individuals was not linked to suicidal ideation. University students are more sensitive to perceptions influenced by the COVID-19 context rather than direct medical facts. This may primarily drive mental health problems. This study provides new information for the governments and policy makers to design effective intervention strategies targeted at reducing the COVID-19 and other infectious diseases.

## Abbreviations

SCM: Stimulus-Cognition-Mental
